# Diagnostic performance of artificial intelligence in oral squamous cell carcinoma detection: a higher-order evidence synthesis through umbrella review and meta-meta-analysis

**DOI:** 10.3389/fdmed.2026.1915105

**Published:** 2026-09-08

**Authors:** Praveen Jodalli, Vineet Vinay, Mahesh Chavan, Ramya Shenoy, Utkarsha Deshpande, Sam Thomas Kuriadom, Apurva Mishra, Raghavendra M. Shetty

**Affiliations:** 1 Department of Public Health Dentistry, Manipal College of Dental Sciences Mangalore, Manipal Academy of Higher Education, Manipal, India; 2 S.T.E.S. Sinhgad Dental College and Hospital, Pune, India; 3 Department of Public Health Dentistry, M.A. Rangoonwala College of Dental Sciences and Research Centre, Pune, India; 4 Department of Clinical Sciences, College of Dentistry, Ajman University, Ajman, United Arab Emirates; 5 Center of Medical and Bio-allied Health Sciences Research, Ajman University, Ajman, United Arab Emirates; 6 Department of Pedodontia and Preventive Dentistry, Sharad Pawar Dental College and Hospital, Datta Meghe Institute of Higher Education and Research (Deemed to be University), Wardha, India

**Keywords:** artificial intelligence, diagnostic accuracy, digital pathology, evidence synthesis, implementation science, meta-review, oral cancer

## Abstract

**Background:**

The integration of AI into oral squamous cell carcinoma (OSCC) diagnostics is a major milestone for digital dentistry and computational pathology, enabling faster, highly accurate detection. While numerous systematic reviews have attempted to synthesize this burgeoning evidence, their conclusions remain discordant and methodological quality varies substantially.

**Aim:**

This umbrella review undertakes a rigorous synthesis of systematic review evidence to establish definitive accuracy metrics, evaluate methodological robustness, quantify evidence redundancy, and delineate critical implementation barriers.

**Methods:**

A comprehensive search was conducted across MEDLINE/PubMed, Embase, Scopus, Web of Science, and Cochrane CENTRAL from inception through September 30, 2025, following PRISMA guidelines and an *a priori* PROSPERO-registered protocol (CRD420251157762). Systematic reviews evaluating AI for OSCC diagnosis were included. Methodological quality was appraised using AMSTAR-2, while primary study overlap was quantified via Corrected Covered Area (CCA). A meta-meta-analysis employing robust variance estimation was performed to pool diagnostic accuracy measures, accounting for statistical dependency.

**Results:**

Fifteen systematic reviews synthesizing 341 primary studies were included. AMSTAR-2 assessment revealed a concerning distribution: only 3 reviews (20%) achieved high confidence, while 3 (20%) were critically low. Moderate primary study overlap was evident (CCA = 9.06%). The meta-meta-analysis, incorporating data from six reviews, yielded a pooled sensitivity of 0.90 (95% CI: 0.81–0.99; I^2^ = 85%; *τ*^2^ = 0.032). The 95% confidence interval suggested that the diagnostic performance of AI may vary substantially in future comparable reviews, reflecting differences in AI models, imaging modalities, study populations, and methodological approaches. Although the pooled estimate indicates high overall sensitivity, the wide confidence and prediction intervals indicate uncertainty regarding the magnitude and consistency of the effect, suggesting that AI performance may not be uniformly reproducible across different clinical settings

**Conclusions:**

AI demonstrated compelling diagnostic accuracy for OSCC, particularly when applied to histopathological specimens. However, the evidence base is compromised by significant heterogeneity, methodological inconsistency, and a critical deficit in implementation research. This synthesis provided an authoritative evidence foundation and a strategic roadmap for researchers, clinicians, and policymakers navigating the evolving landscape of AI in oral oncology.

**Clinical relevance:**

The integration of AI into routine clinical practice may assist clinicians in identifying suspicious lesions at earlier stages, improving diagnostic consistency, reducing inter-observer variability, and supporting timely referral and treatment decisions. Furthermore, AI-driven tools can facilitate large-scale screening programs, particularly in resource-constrained settings where access to specialist expertise may be limited. Although promising, successful clinical implementation requires rigorous validation, standardization of algorithms, transparency in decision-making processes, and evaluation of real-world effectiveness to ensure safe, reliable, and equitable patient care.

**Systematic Review Registration:**

PROSPERO [CRD420251157762].

## 1 Introduction

Oral squamous cell carcinoma (OSCC) constitutes a significant global health burden, with over 370,000 new cases and approximately 180,000 deaths annually worldwide (1). The prognosis of OSCC remains heavily stage-dependent, with five-year survival rates plummeting from 80%–90% for localized disease to less than 30% for metastatic presentations (2). This stark survival disparity underscores the critical importance of early detection, yet current diagnostic paradigms—reliant on visual-tactile examination followed by invasive biopsy and histopathological assessment—are plagued by subjectivity, inter-observer variability, and limited accessibility in resource-constrained settings (3, 4).

The advent of artificial intelligence (AI), particularly through advances in machine learning (ML) and deep learning (DL), has catalyzed a transformative shift across medical diagnostics (5). In oral oncology, AI applications have proliferated across multiple domains: convolutional neural networks (CNNs) analyzing whole-slide histopathology images for automated detection and grading (6); computer vision algorithms interpreting clinical photographs for community-based screening (7); and AI-enhanced optical coherence tomography (OCT) providing sub-epithelial visualization for early invasion assessment (8).

This rapid expansion of primary research has generated a corresponding proliferation of secondary evidence syntheses. Systematic reviews and meta-analyses have emerged as essential tools for consolidating findings, yet their conclusions are frequently discordant (9). Variations in search strategies, inclusion criteria, risk of bias assessments, and statistical methodologies contribute to this inconsistency (10). This creates a fragmented evidence landscape that challenges clinicians, health technology assessors, and policymakers seeking to derive actionable guidance.

An umbrella review, defined as a systematic review of systematic reviews, represents the comprehensive synthesis in the evidence-based medicine hierarchy (11). By integrating findings across multiple systematic reviews, it can resolve inconsistencies, provide definitive quantitative summaries, critically appraise methodological quality across the entire evidence base, quantify redundancy, and identify unequivocal research gaps that individual reviews may not capture (12). To date, no such comprehensive synthesis exists for AI applications in OSCC diagnosis, leaving a critical knowledge void at the intersection of computational science and clinical oral oncology.

This umbrella review was therefore undertaken with the following specific objectives:

1. To systematically identify, appraise, and synthesize all systematic reviews and meta-analyses evaluating the diagnostic accuracy of AI technologies for OSCC.
2. To quantitatively pool accuracy estimates through meta-meta-analysis while statistically accounting for primary study overlap.
3. To critically evaluate the methodological quality of existing systematic reviews using the AMSTAR-2 framework and assess the overall certainty of evidence using GRADE.
4. To provide consolidated, evidence-based recommendations for clinical practice, health policy, and future research directions in AI-enhanced oral cancer diagnostics.

## 2 Methodology

### 2.1 Protocol and registration

This investigation was conducted following an *a priori* protocol that was prospectively registered with the International Prospective Register of Systematic Reviews (PROSPERO) on December 20, 2025 (Registration ID: CRD420251157762). The methodology adheres to the Joanna Briggs Institute (JBI) guidelines for umbrella reviews (13) and is reported in accordance with the Preferred Reporting Items for Systematic Reviews and Meta-Analyses (PRISMA) 2020 statement (14). Any deviations from the registered protocol are explicitly documented and justified in the Supplementary Materials.

### 2.2 Eligibility criteria

We employed an extended PICOS (Population, Intervention, Comparator, Outcomes, Study design, Setting) framework to define eligibility with precision:

Population: Systematic reviews and/or meta-analyses focusing on the diagnosis of histologically confirmed oral squamous cell carcinoma (OSCC) in human populations. Reviews including oral potentially malignant disorders (OPMDs) were considered only if they reported separate, extractable diagnostic accuracy data specifically for OSCC.

Intervention/Index Test: Any artificial intelligence-based diagnostic technology, including:

(a) Traditional machine learning algorithms (e.g., Support Vector Machines, Random Forests, k-Nearest Neighbors)
(b) Deep learning architectures (e.g., Convolutional Neural Networks [CNNs] including ResNet, VGG, DenseNet, EfficientNet; Recurrent Neural Networks; Transformers)
(c) Hybrid AI models combining multiple approaches
(d) AI applications to any relevant data modality: histopathology (whole slide images, tissue microarrays), clinical or photographic images, optical coherence tomography (OCT), autofluorescence imaging, cytology, radiography (CBCT, panoramic), or hyperspectral imaging.

Comparator/Reference Standard: The histological diagnosis of OSCC based on biopsy or surgical specimen examination by a qualified pathologist served as the primary reference standard. Comparisons with expert clinical examination or conventional non-AI diagnostic methods were also considered.

Outcomes: Primary outcomes comprised diagnostic accuracy metrics: sensitivity, specificity, diagnostic odds ratio (DOR), area under the receiver operating characteristic curve (AUC), and positive/negative likelihood ratios (PLR/NLR). Secondary outcomes included measures of clinical utility, implementation feasibility (e.g., integration time, user acceptability), economic evaluations, and reported barriers to adoption.

Study Designs: Only completed, peer-reviewed systematic reviews and/or meta-analyses were eligible. Preprints, conference abstracts, editorials, and other non-peer-reviewed publications were excluded. To qualify, a review must have described a comprehensive, reproducible search strategy across at least two electronic bibliographic databases and presented explicit inclusion and exclusion criteria. We excluded protocol papers, scoping reviews, narrative reviews, editorial commentaries, conference abstracts without associated full manuscripts, and primary research studies. To mitigate publication bias and capture the most current evidence, preprints from established servers (medRxiv, arXiv, bioRxiv) reporting completed systematic reviews were also considered.

Setting: No restrictions were placed on clinical setting (e.g., hospital, primary care, community screening) or geographical location.

### 2.3 Information sources and search strategy

A comprehensive, systematic literature search was designed in collaboration with an information specialist and executed across five major international bibliographic databases from their inception through September 30, 2025:

1. MEDLINE via PubMed
2. Embase via Ovid
3. Scopus
4. Web of Science Core Collection
5. Cochrane Central Register of Controlled Trials (CENTRAL)

The search strategy employed a combination of controlled vocabulary (Medical Subject Headings [MeSH] in PubMed, Emtree terms in Embase) and free-text keywords. The strategy was structured around three core conceptual blocks:

1. Oral Cancer Concepts: (“Mouth Neoplasms”[Mesh] OR “Oral Cancer” OR “Oral Squamous Cell Carcinoma” OR “OSCC” OR “oral cavity cancer” OR “tongue neoplasms”)
2. Artificial Intelligence Concepts: (“Artificial Intelligence”[Mesh] OR “Machine Learning”[Mesh] OR “Deep Learning” OR “Neural Networks, Computer” OR “Computer-Assisted Diagnosis” OR “convolutional neural network” OR “CNN” OR “support vector machine” OR “SVM”)
3. Systematic Review Concepts: (“Systematic Review”[Publication Type] OR “Meta-Analysis”[Publication Type] OR “umbrella review” OR “review of reviews” OR “systematic review” [tiab])

Boolean operators (AND, OR, NOT) were tailored to each database's syntax. No language restrictions were applied initially, though only English-language reviews were ultimately included due to resource constraints. The strategies for all databases are available in Supplementary File S1.

To ensure comprehensiveness and minimize retrieval bias, we implemented several complementary search strategies such as Backward citation searching of reference lists from all included reviews and forward citation searching via Google Scholar for articles citing key included reviews, Systematic searches of preprint servers (medRxiv, arXiv, bioRxiv) and dissertation databases (ProQuest Dissertations & Theses Global), Contact with corresponding authors of included reviews and leading researchers in the field to identify additional relevant or forthcoming work, Manual review of tables of contents from key journals in oral oncology, pathology, and medical AI published in the last six months of the search period.

### 2.4 Study selection process

All retrieved records were imported into the Rayyan web application for systematic reviews for deduplication and management. The selection process was conducted independently by two reviewers (V.V. and P.J.) in two sequential phases. Firstly, Title and Abstract Screening: Reviewers screened all records against the eligibility criteria. A liberal approach was taken at this stage—any record deemed potentially relevant by either reviewer proceeded to full-text review. Secondly, Full-Text Assessment: Full-text articles of all potentially eligible records were obtained and independently evaluated by the same two reviewers against the complete PICOS criteria. Reasons for exclusion were documented for each excluded article using a standardized categorization system. Disagreements at either stage were resolved through discussion between the two reviewers. If consensus could not be reached, a third senior reviewer (R.S.) arbitrated the final decision. The entire study selection process was documented using a PRISMA 2020 flow diagram.

### 2.5 Data extraction

Data from each included systematic review were extracted independently by two reviewers (M.C. and U.D.) using a standardized, pilot-tested data extraction form. The form captured bibliographic and Descriptive Data such as First author, publication year, journal, Digital Object Identifier (DOI), review type (systematic review, meta-analysis, or both), funding sources, conflicts of interest. Methodological Characteristics such as number of primary studies included, databases searched, search date range, eligibility criteria as stated, methods for study selection and data extraction, approach to quality/risk of bias assessment were extracted. Intervention details, outcome data and synthesis methods used for narrative and quantitative synthesis, approaches to heterogeneity assessment and exploration, publication bias assessment were also extracted. Lastly, key Findings such as authors' synthesized results, reported limitations, and conclusions regarding clinical applicability and future research needs were extracted.

Following independent extraction, the two reviewers compared their completed forms. Discrepancies were resolved by referring back to the original publication and through consensus discussion. A senior reviewer (V.V.) performed final verification of the complete extraction dataset. Inter-rater reliability for data extraction was assessed using Cohen's kappa statistic.

### 2.6 Critical appraisal of methodological quality

The methodological quality and risk of bias of each included systematic review were independently assessed by two reviewers (R.S. and U.D.) using the AMSTAR-2 (A MeaSurement Tool to Assess systematic Reviews 2) checklist (15). AMSTAR-2 is a 16-item critical appraisal tool for systematic reviews that includes 7 domains considered critical to the validity of a review's conclusions. Each review was rated for each domain as “Yes,” “Partial Yes,” or “No.” Based on weaknesses in the critical domains, the overall confidence in the results of each review was classified as: High -No or one non-critical weakness, Moderate—More than one non-critical weakness, Low: One critical flaw with or without non-critical weaknesses, Critically Low—More than one critical flaw. Inter-rater agreement was calculated using Cohen's kappa, with disagreements resolved through discussion or arbitration by a third reviewer (V.V.).

### 2.7 Assessment of primary study overlap

A significant methodological challenge in umbrella reviews is the inclusion of the same primary study in multiple systematic reviews, creating statistical dependency that can bias meta-meta-analysis results. We quantified this overlap using the Corrected Covered Area (CCA) metric, as proposed by Pieper et al. (16).

Theformulausedwas:CCA=(N−r)/(rc−r)

Where: *N* = total number of inclusions of primary studies across all reviews (counting each primary study each time it appears in any review); *r* = number of reviews included in our umbrella review; c = number of unique primary studies across all reviews.

Interpretation followed established thresholds (16): Slight overlap: CCA 0%–5%; Moderate overlap: CCA 6%–10% (our finding); High overlap: CCA 11%–15%; Very high overlap: CCA >15%

To calculate CCA, we extracted the list of included primary studies from each review where available. When not fully reported, we estimated based on the methodology described and the reported number of studies.

### 2.8 Data synthesis strategy

#### 2.8.1 Narrative synthesis

A structured narrative synthesis was conducted first, organized thematically around the review objectives. We employed the “best fit” framework synthesis approach (17), using the WHO's health technology assessment framework as an organizing structure. Findings were synthesized regarding: diagnostic performance characteristics, comparisons with conventional methods, identified technical and clinical limitations, reported implementation barriers and facilitators, and evidence gaps. Consistency and discordance across reviews were explicitly highlighted.

#### 2.8.2 Quantitative synthesis (meta-meta-analysis)

A quantitative synthesis was undertaken only for systematic reviews that reported pooled diagnostic accuracy estimates generated through formal meta-analysis. Meta-meta-analysis was selected because the objective of this umbrella review was to quantitatively synthesize evidence from systematic reviews rather than individual primary studies. Reviews were excluded from the meta-meta-analysis if they (i) presented only narrative findings without pooled estimates, (ii) evaluated outcomes that were not directly comparable with the predefined outcome of interest, or (iii) did not provide sufficient statistical data for re-analysis. These reviews remained part of the qualitative synthesis, methodological quality assessment, and evidence mapping. Summary estimates of sensitivity and specificity were logit-transformed for analysis to stabilize variances and normalize distributions (18). The pooled estimates and corresponding 95% confidence intervals were subsequently back-transformed to the original proportion scale, thereby constraining all summary estimates between 0 and 1. Random-effects models using restricted maximum likelihood (REML) estimation were employed, as substantial between-review heterogeneity was anticipated.

Analyses were conducted using R statistical software (version 4.3.0) with the metafor, meta, and dmetar packages. Heterogeneity was quantified using the I^2^ statistic (with 25%, 50%, and 75% representing low, moderate, and high heterogeneity) and *τ*^2^ (the between-study variance). Prediction intervals were calculated alongside 95% confidence intervals to provide a range within which the effect size of a new review would fall, given the observed heterogeneity (19). To account for the statistical dependency introduced by overlapping primary studies, we employed robust variance estimation (RVE) with small-sample corrections using the club Sandwich package in R (20–22). This method provides valid inference even when effect size estimates are correlated.

### 2.9 Certainty of evidence assessment

The overall certainty (quality) of the body of evidence for each key outcome was assessed using the GRADE (Grading of Recommendations, Assessment, Development, and Evaluations) framework adapted for diagnostic test accuracy studies (23–25). Two reviewers (V.V. and P.J.) independently rated evidence certainty as High, Moderate, Low, or Very Low based on five domains:

1. Risk of bias: Informed by AMSTAR-2 assessments of the included reviews and reported risk of bias in primary studies.
2. Inconsistency: Statistical heterogeneity (I²) and qualitative differences in direction of effect.
3. Indirectness: Applicability to the review question, population, intervention, and outcomes of interest.
4. Imprecision: Width of confidence intervals and whether they cross clinically important thresholds.
5. Publication bias: As assessed by funnel plots, Egger's test, and consideration of grey literature.

We developed specific guidance for applying GRADE to umbrella reviews, particularly regarding how to assess risk of bias when the unit of analysis is a systematic review rather than a primary study. Discrepancies in ratings were resolved through consensus discussion.

## 3 Results

### 3.1 Study selection

**Figure 1:**
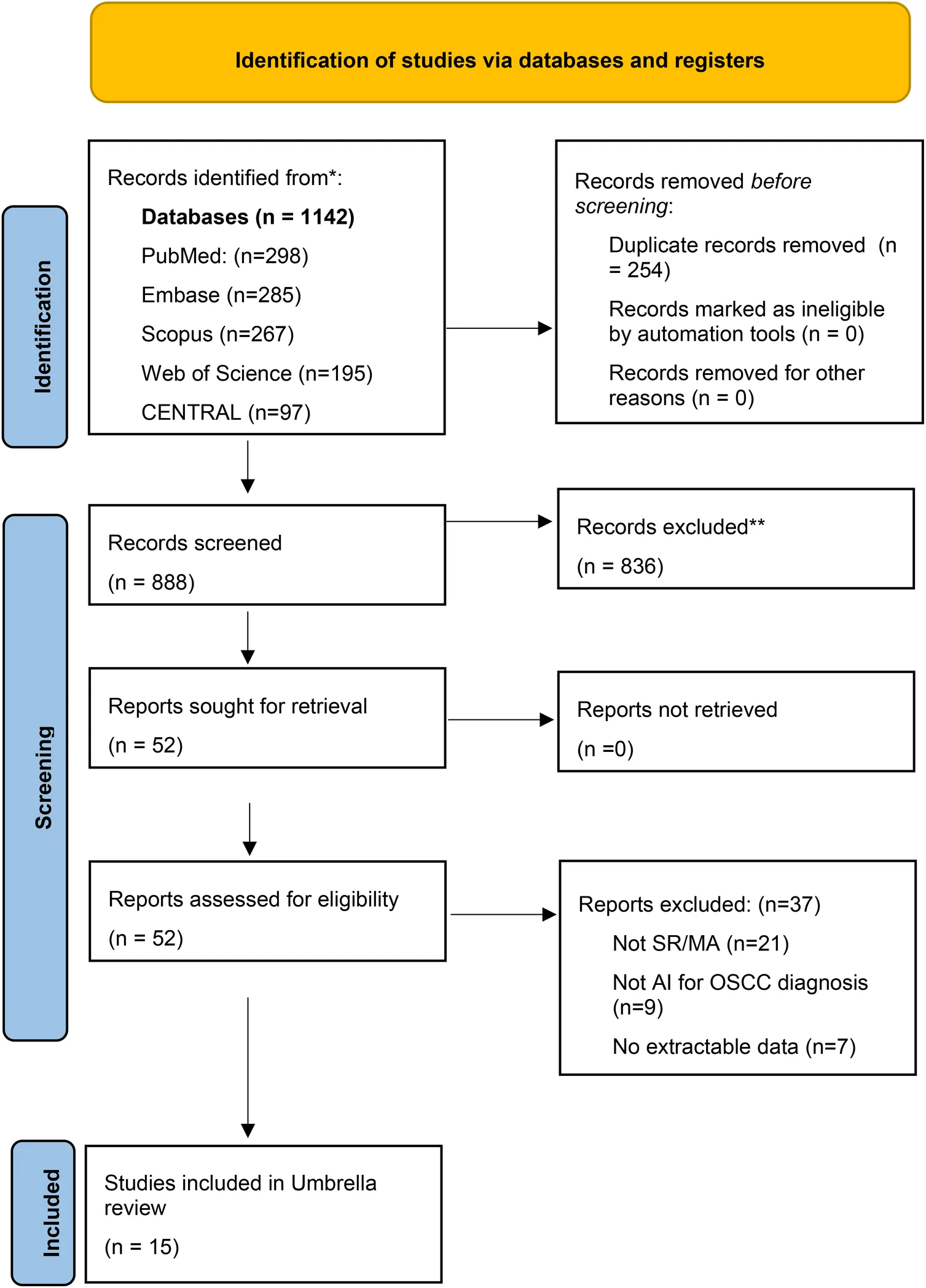
PRISMA 2020 flow diagram for study selection.

The PRISMA 2020 flow diagram (Figure 1) illustrates the study selection process. Our systematic search identified 1,142 records from database searching and 33 additional records from other sources. After removal of 254 duplicates, 888 unique records underwent title and abstract screening. Of these, 836 were excluded as clearly irrelevant. The full texts of the remaining 52 articles were retrieved and assessed for eligibility. Thirty-seven articles were excluded with reasons: 21 were not systematic reviews/meta-analyses, 09 did not focus on AI for OSCC diagnosis, 7 had no extractable diagnostic accuracy data, and 3 were non-English publications. Ultimately, 15 systematic reviews met all eligibility criteria and were included in this umbrella review.

### 3.2 Characteristics of included systematic reviews

Table 1 presents the comprehensive characteristics of the included systematic reviews (26–40), published between 2020 and 2025. Collectively, these reviews synthesized evidence from 341 primary studies (range: 8 to 55 studies per review, with one review not specifying the exact number). The temporal distribution shows increasing publication frequency: 1 review in 2020, 2 in 2021, 2 in 2022, 1 in 2023, 6 in 2024, and 3 in 2025, reflecting the accelerating interest in this field. Seven reviews (46.7%) were systematic reviews only, while eight (53.3%) included meta-analysis. Among meta-analyses, five conducted bivariate or hierarchical models appropriate for diagnostic accuracy data.

**Table 1:** Study characteristics.

First author	Year	Journal name	Study type	Number of primary studies included	Database(s) searched	Type of AI model used	AI model types applied to OSCC	Image modality used	OSCC-specific outcomes assessed	Risk of bias/quality assessment tool	Outcomes assessed	Results
Mahmood et al. (26)	2020	*Oral Oncology*	Systematic review	11	MEDLINE (OVID), Scopus, Web of Science	ML, DL	SVM, k-NN, ANN, CNN, segmentation & classification models	Histopathology (WSI)	Detection and grading of OSCC, dysplasia	QUADAS-2 (tailored)	Accuracy, sensitivity, specificity	Accuracy ranged 79%–100%; evidence low quality, high risk of bias
Alabi et al. (27)	2021	*Virchows Archiv*	Systematic review	41	Ovid MEDLINE, PubMed, Scopus, Web of Science, IEEE	Machine learning models (primarily SVM, ANN; also, ensemble methods in included studies)	ML models mainly SVM, ANN, ensemble models	Histopathology images, clinical data, imaging (varied across studies)	OSCC diagnosis and prognosis; sensitivity (0.70–1.00), specificity (0.57–1.00), accuracy (63.4%–100%), survival prediction, recurrence prediction, nodal metastasis prediction	PROBAST (Prediction model Risk of Bias Assessment Tool)	Diagnostic accuracy, prognostic prediction (sensitivity, specificity, accuracy, AUC) in OSCC	OSCC diagnosis and prognosis; sensitivity (0.70–1.00), specificity (0.57–1.00), accuracy (63.4%–100%), survival prediction, recurrence prediction, nodal metastasis prediction
Khanagar et al. (28)	2021	*Diagnostics*	Systematic review	16	PubMed, Google Scholar, Scopus, Embase, Cochrane, Web of Science, Saudi Digital Library	ANN, ML algorithms (including SVM, regression-based ML in included studies)	ANN, SVM, ML predictive models	Fluorescence imaging, hyperspectral images, cytology, CT images	OSCC diagnosis accuracy; prediction of OSCC occurrence and prognosis using clinical and biomarker data	QUADAS-2	Diagnosis of oral cancer and prediction of prognosis (accuracy, sensitivity, specificity)	OSCC diagnosis accuracy; prediction of OSCC occurrence and prognosis using clinical, demographic, and biomarker data
Elmakaty et al. (29)	2022	*Critical Reviews in Oncology/Hematology*	Systematic review & meta-analysis	16	PubMed, Embase, Scopus, Cochrane Library, ProQuest, Web of Science	Multiple AI models (ML and DL; CNNs, SVMs, ANNs among included studies)	Multiple AI models (CNNs, SVMs, ANNs)	Histopathology images, clinical imaging	Diagnostic accuracy for OSCC vs. histopathology; pooled sensitivity 92.0%, specificity 91.9%, DOR 132	QUADAS-2	Diagnostic accuracy (sensitivity, specificity, DOR, likelihood ratios, AUC) for OSCC detection	Diagnostic accuracy for OSCC detection vs. histopathology; pooled sensitivity 92.0%, specificity 91.9%, DOR 132
Kim et al. (30)	2022	*Cancers*	Systematic review & meta-analysis	19	PubMed, Embase, Web of Science, Scopus, Cochrane Central, Google Scholar	AI image-analysis models (CNN-based systems applied to photos, autofluorescence, OCT)	CNN-based AI image analysis	Oral photographs, autofluorescence imaging, OCT	OSCC discrimination from normal mucosa; DOR 121.66; sensitivity ∼92%; OCT-AI highest accuracy	QUADAS-2	Diagnostic discrimination of cancerous vs. normal/precancerous mucosa (sensitivity, specificity, DOR, AUC)	Discrimination of OSCC from normal mucosa; diagnostic odds ratio 121.66; sensitivity ∼92%; OCT-AI highest OSCC diagnostic accuracy
Khanagar et al. (31)	2023	*Biomedicines*	Systematic review	19	PubMed, Scopus, Embase, Cochrane Library, Web of Science, Google Scholar, Saudi Digital Library	ML and DL models (CNNs, ANN-based classifiers on histopathology images)	CNNs and ANN-based DL models on histopathology	Histopathological images	OSCC diagnosis, grading, and prognosis prediction; accuracy 89.47%–100%, sensitivity 97.76–99.26%, specificity 92–99.42%	QUADAS-2, GRADE	Diagnostic accuracy, classification, grading, and prognosis prediction of oral cancer	OSCC diagnosis, grading, and prognosis prediction; accuracy 89.47%–100%, sensitivity 97.76–99.26%, specificity 92–99.42%
Jerjes et al. (32)	2024	*Journal of Clinical Medicine*	Systematic review	9	PubMed, Embase, Scopus, Google Scholar, Cochrane Central, Web of Science	AI-assisted OCT algorithms (ML- and DL-based image interpretation)	AI-assisted OCT algorithms (ML/DL image interpretation)	Optical Coherence Tomography (OCT)	OSCC detection accuracy; sensitivity 75%–100%, specificity 71%–100%; AI-augmented OCT superior	Not explicitly stated (quality assessment performed, tool not clearly named)	Diagnostic accuracy of OCT with AI (sensitivity, specificity) for oral cancer detection	OSCC detection accuracy using OCT; sensitivity 75%–100%, specificity 71%–100%; improved performance with AI-augmented OCT
Li et al. (33)	2024	*International Journal of Surgery*	Systematic review & meta-analysis	-	PubMed, Embase, Web of Science, Scopus	ML and DL imaging-based AI models	ML and DL imaging-based models	Clinical photography, autofluorescence, OCT	OSCC detection accuracy; sensitivity ∼90%, specificity ∼89%, AUC up to 0.94	Not explicitly stated (methodological quality assessed, tool not clearly specified)	Diagnostic accuracy of AI for OPMD and oral cancer (sensitivity, specificity, DOR, SROC/AUC)	OSCC detection accuracy within OPMD spectrum; sensitivity ∼90%, specificity ∼89%, AUC up to 0.94; best performance with clinical photography AI
Malhotra et al. (34)	2024	*Asian Pac J Cancer Prev*	Systematic review & meta-analysis	14	PubMed, Google Scholar, EBSCOhost	AI (ML & DL)	CNN, ANN, hybrid models	Histopathology images	Early OSCC detection	QUADAS-2	Sensitivity, specificity, DOR, AUC	Pooled sensitivity 0.43, specificity 0.50; poor–moderate diagnostic accuracy
Pirayesh et al. (35)	2024	*J Oral Pathol Med*	Systematic review & meta-analysis	17	PubMed, Scopus, Embase, IEEE, Google Scholar, ArXiv	Deep learning	CNN, FCN, U-Net, segmentation & classification models	Histopathology (WSI, TMA)	OSCC detection & segmentation	QUADAS-2	Accuracy, sensitivity, specificity, AUC	Pooled sensitivity 0.98, specificity 0.93; high diagnostic accuracy
Sahoo et al. (36)	2024	*Frontiers in Oral Health*	Systematic review & meta-analysis	55 (18 meta-analysis)	PubMed, Scopus, IEEE	ML & DL	CNN, hybrid DL models	Histopathology, photographic, radiologic images	OSCC & OPMD detection	QUADAS-2	Sensitivity, specificity, DOR, AUC	Sensitivity 0.87, specificity 0.81, AUC 0.97; DL superior
Thakuria et al. (37)	2024	*Expert Rev Med Devices*	Systematic review	19	PubMed, Scopus, Google Scholar	Deep learning	CNN-based models	Smartphone & DSLR clinical images	Early OSCC detection	Narrative quality appraisal	Accuracy, sensitivity, feasibility	High diagnostic potential for low-resource screening; external validation lacking
Chowdhury et al. (38)	2025	*Cureus*	Systematic review	11	PubMed, Scopus, Web of Science, CINAHL	Machine learning and deep learning models (CNNs, ensemble learning, attention-based models)	ML and DL models, predominantly CNN-based image classifiers	Clinical oral photographs, histopathology images	Diagnostic accuracy of AI for OSCC detection; sensitivity >85%, accuracy >90%	QUADAS-2	Diagnostic performance of AI for OSCC detection (sensitivity, specificity, accuracy)	Diagnostic accuracy of AI for OSCC detection using oral images; sensitivity >85%, accuracy >90% in most OSCC studies
Nieri et al. (39)	2025	*Oral Diseases*	Comparative test-accuracy SR	8	PubMed, EMBASE, Scopus, Google Scholar, ClinicalTrials.gov	Deep learning only	CNN architectures (ResNet, VGG, DenseNet, EfficientNet)	Clinical photographic images	OSCC vs. non-OSCC classification	QUADAS-C	Sensitivity, specificity	DL comparable to experts; better sensitivity than postgraduate students
Ren et al. (40)	2025	*J Stomatol Oral Maxillofac Surg*	Systematic review & meta-analysis	24	PubMed, Scopus, Web of Science, Embase, Google Scholar	ML & DL	CNN, SVM, ANN	Histopathology & clinical images	OSCC detection	QUADAS-2	Sensitivity, specificity, PLR, NLR, DOR	Sensitivity **0.95**, specificity 0.95; high heterogeneity

The median number of databases searched was 5 (range: 3–7). The most commonly searched databases were PubMed (100%), Scopus (87%), and Web of Science (73%). Only three reviews (20%) reported searching preprint servers or grey literature. Twelve reviews (80%) employed a standardized tool for assessing risk of bias/quality of primary studies. QUADAS-2 was the most frequently used tool (9 reviews, 60%), followed by PROBAST (1 review), QUADAS-C (1 review), and narrative appraisal (1 review). Three reviews did not clearly specify their assessment tool. Histopathology-based AI was the most studied modality (9 reviews, 60%), followed by clinical photography (6 reviews, 40%), OCT (3 reviews, 20%), and mixed/other modalities (5 reviews, 33%). Deep learning approaches (particularly CNNs) dominated the recent literature, featured in 12 reviews (80%). Traditional machine learning models were the focus in 3 earlier reviews (20%).

### 3.3 Methodological quality assessment

The results of the AMSTAR-2 assessment are presented in Table 2. The methodological quality of included reviews exhibited considerable variability, with an overall mean AMSTAR-2 score of 9.1 out of 16 (range: 4–15).

**Table 2:** Quality assessment of included reviews using AMSTAR-2 tool.

Review (Year)	1. Protocol	2. Duplicate Study Selection	3. Comprehensive Search	4. Status of Publication	5. List of Studies	6. Characteristics	7. Critical Appraisal	8. RoB in Conclusions	9. Appropriate Meta-Analysis Methods	10. RoB in Meta-Analysis	11. RoB in Narrative Synthesis	12. Funding Sources	13. Explanation of Heterogeneity	14. Investigation of Publication Bias	15. Conflict of Interest	16. Overall Rating
Mahmood et al. (26)	PY	Y	Y	Y	Y	Y	N	Y	N	N/A	N	N	Y	N	Y	Low
Alabi et al. (27)	Y	PY	Y	Y	Y	Y	N	Y	PY	Y	Y	N	Y	N	Y	Moderate
Khanagar et al. (28)	Y	Y	Y	Y	Y	Y	N	Y	PY	N/A	N	N	Y	N	Y	Low
Elmakaty et al. (29)	Y	Y	Y	Y	Y	Y	Y	Y	Y	Y	Y	N	Y	Y	Y	High
Kim et al. (30)	Y	Y	Y	Y	Y		Y	Y	Y	Y	Y	Y	N	Y	Y	High
Khanagar et al. (31)	Y	Y	Y	Y	Y	Y	N	Y	Y	Y	Y	N	Y	Y	Y	Moderate
Jerjes et al. (32)	PY	Y	Y	Y	Y	Y	N	N	N	N/A	N	N	Y	N	Y	Critically Low
Li et al. (33)	Y	Y	Y	Y	Y	Y	N	Y	Y	Y	Y	N	Y	N	Y	Moderate
Malhotra et al. (34)	N	Y	Y	Y	Y	Y	N	N	N	N/A	N	N	Y	N	Y	Critically Low
Pirayesh et al. (35)	Y	Y	Y	Y	Y	Y	N	Y	Y	Y	Y	N	Y	Y	Y	Moderate
Sahoo et al. (36)	Y	Y	Y	Y	Y	Y	Y	Y	Y	Y	Y	N	Y	Y	Y	High
Thakuria et al. (37)	N	Y	PY	Y	Y	Y	N	N	N	N/A	N	N	Y	N	Y	Critically Low
Chowdhury et al. (38)	Y	Y	Y	Y	Y	Y	N	Y	N	N/A	N	N	Y	N	Y	Low
Nieri et al. (39)	Y	Y	Y	Y	Y	Y	PY	Y	N	N/A	PY	N	Y	N	Y	Moderate
Ren et al. (40)	Y	Y	Y	Y	Y	Y	N	Y	PY	N/A	N	N	Y	N	Y	Low

N, No; Y, Yes; PY, Partially Yes; N/A, Not applicable; N, Not mentioned.

Three reviews (29, 32, 36) (20%) showed high methodological quality. These reviews demonstrated comprehensive methodology, appropriate meta-analysis techniques, thorough risk of bias assessment, and investigation of publication bias. Moderate confidence was observed in 5 reviews (27, 31, 33, 35, 39) (33.3%). These reviews had generally sound methods but exhibited weaknesses in non-critical domains, most commonly in providing a list of excluded studies or reporting funding sources. Low confidence was observed in 4 reviews (26.7%) (26, 28, 38, 40). These reviews had one critical flaw, typically in the risk of bias assessment of included studies (Item 9) or in the justification for excluding individual studies (Item 7). Jerjes et al. (31), Malhotra et al. (34), Thakuria et al. (37) showed critically low confidence. These reviews had multiple critical flaws, often including inadequate risk of bias assessment, inappropriate synthesis methods, and failure to account for risk of bias in interpreting results.

The most prevalent critical weaknesses across all reviews were:

1. Item 7 (Justification for excluded studies): 13 reviews (86.7%) did not provide a list of excluded studies with justifications.
2. Item 9 (Risk of bias assessment): 9 reviews (60.0%) used inadequate methods or did not perform risk of bias assessment appropriately.
3. Item 11 (Appropriate meta-analysis methods): Among the 8 reviews conducting meta-analysis, 2 (25%) used inappropriate methods for diagnostic accuracy data.

Inter-rater agreement for AMSTAR-2 assessments was substantial (Cohen's *κ* = 0.78, 95% CI: 0.65–0.91). All discrepancies were resolved through consensus discussion among the authors. The details of methodological quality assessment are mentioned in Supplementary File S2.

### 3.4 Assessment of primary study overlap

We calculated the Corrected Covered Area (CCA) to quantify redundancy in the evidence base. Based on extraction of primary study lists where available and estimation where lists were not available, we identified approximately 241 unique primary studies across the 15 reviews, with a total of 341 inclusions (counting duplicates). Applying the CCA formula:

CCA=(341−15)/(15×241−15)=326/3600≈0.0906"or"9.06%

A CCA of 9.06% indicated moderate overlap according to established thresholds. This suggests that while there is meaningful redundancy in the systematic review literature (the same primary studies appearing in multiple reviews), it is not excessive. The temporal pattern showed increasing overlap in more recent reviews (2024–2025) as the field consolidates around key studies and datasets.

### 3.5 Narrative synthesis of key findings

#### 3.5.1 Diagnostic performance across modalities

The included reviews consistently reported high diagnostic accuracy for AI systems in OSCC detection, though with substantial variability in reported ranges:

Histopathology-Based AI reported sensitivity ranged from 70%–100%, specificity from 57%–100%, and accuracy from 63.4%–100% across different primary studies (15). Recent, high-quality meta-analyses reported pooled estimates as high as sensitivity 0.98 (95% CI: 0.96–0.99) and specificity 0.93 (0.90–0.95) (27). DL models, particularly CNNs (ResNet, VGG, DenseNet architectures), consistently outperformed traditional ML approaches. Applications extended beyond detection to grading (differentiating well/moderately/poorly differentiated OSCC) and segmentation (delineating tumor boundaries) in some reviews (28–30).

Clinical Photography-Based AI reported sensitivity typically exceeded 85%, with accuracy often surpassing 90% in screening applications (29). Performance was comparable to expert oral medicine specialists and superior to less experienced clinicians or postgraduate students (30). Mobile and smartphone-based implementations showed particular promise for low-resource screening but lacked external validation (31). Key challenges included variability in lighting conditions, image quality, and angulation across different clinical settings.

OCT-Based AI reported sensitivity of 75%–100% and specificity of 71%–100% for detecting early sub-epithelial invasion (32). AI augmentation consistently improved the diagnostic accuracy of conventional OCT interpretation. OCT-AI showed potential for non-invasive assessment of surgical margins but evidence was limited to small feasibility studies. High equipment costs and technical expertise requirements were noted as significant implementation barriers.

#### 3.5.2 Comparison with conventional diagnostic methods

All reviews that included direct comparisons found AI systems were either comparable to or exceeded conventional diagnostic approaches. For histopathology interpretation, several reviews concluded AI systems could match the diagnostic accuracy of expert pathologists for OSCC detection and grading, with the potential to reduce inter-observer variability and diagnostic time (32). For clinical screening, AI systems generally surpassed visual examination by non-specialists and showed particular promise for triage in low-resource, high-burden settings (34). For OCT interpretation, AI augmentation provided more consistent and quantitative assessment compared to subjective visual interpretation by clinicians.

A recurrent limitation highlighted across reviews was that most comparative studies were retrospective, with limited prospective validation in real-world clinical workflows. Only three reviews mentioned any prospective studies, and these were typically single-center with small sample sizes.

#### 3.5.3 Technical and methodological limitations identified by primary studies

1. Thematic analysis of the limitation's sections revealed consistent concerns:

Data limitations: Small, single-center, retrospective datasets with potential selection bias were cited in 12 reviews (80%). Lack of large, diverse, multicenter datasets for training and validation was a universal concern (35). Technical heterogeneity: Considerable variation in image acquisition protocols, preprocessing methods, annotation standards, and AI architectures across studies limited comparability and generalizability (36) Validation gaps: Limited external validation on independent datasets and minimal prospective testing in clinical settings were noted in 11 reviews (73.3%). Most studies used internal validation with data splitting rather than true external validation (37). Algorithmic transparency: The “black box” nature of complex deep learning models was identified as a barrier to clinical trust and adoption in 8 reviews (53.3%). Only two reviews mentioned studies investigating explainable AI (XAI) techniques for OSCC diagnosis (38). Reporting incompleteness: Inconsistent reporting of confidence intervals, training-testing splits, calibration metrics, and computational requirements hampered evidence synthesis.

2. Evidence Gaps identified by primary studies

Analysis of conclusions and future research sections identified consistent, cross-cutting evidence gaps such as:

a) Prospective Clinical Trials: A critical need for well-designed, multicenter, prospective studies validating AI tools against current standards of care with clinically relevant endpoints.
b) Implementation Science: An almost complete absence of research on practical implementation aspects: workflow integration, cost-effectiveness, clinician training, change management, and long-term monitoring of algorithm performance in practice (41).
c) Health Equity Research: Limited evidence on performance and applicability in diverse global populations, particularly in low-resource settings. Need for deliberately diverse training datasets and fairness-aware algorithm development (42).
d) Standardization: Lack of consensus on optimal validation protocols, performance benchmarks, and reporting standards (adaptation of STARD-AI, TRIPOD-AI guidelines) (43).
e) Longitudinal Studies: No studies identified assessing the long-term impact of AI implementation on patient outcomes, healthcare utilization, or population-level oral cancer burden.

### 3.6 Meta-meta-analysis results

Although fifteen systematic reviews were included in the umbrella review, only six (29, 30, 33, 35, 36, 40) provided pooled diagnostic accuracy estimates together with sufficient statistical information required for quantitative synthesis. The remaining reviews were excluded from the meta-meta-analysis because they were narrative systematic reviews (26–28, 31, 32, 37–39) and two reviews lacked sufficient statistical information required for quantitative synthesis (34, 40).

**Figure 2:**
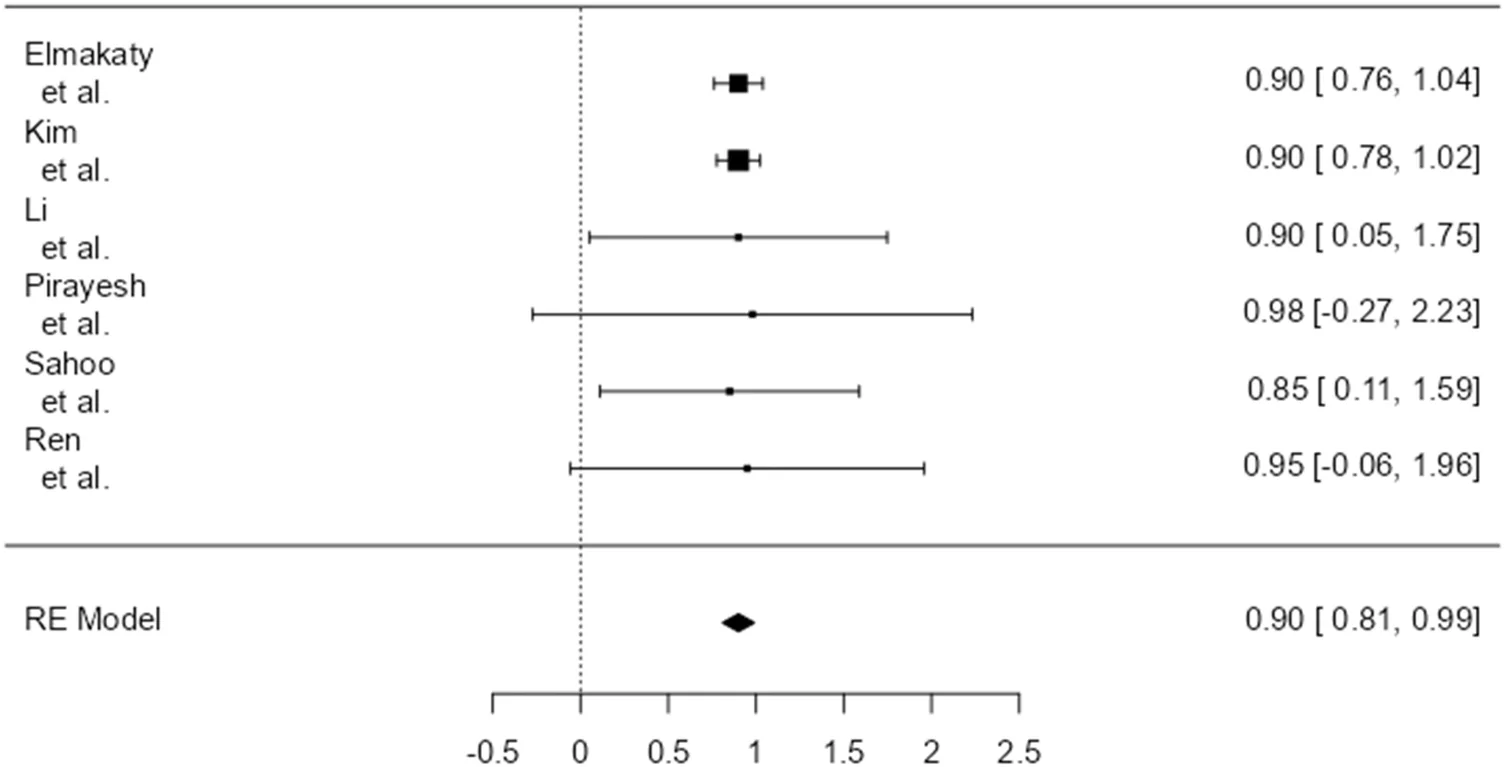
Forest plot for pooled sensitivity.

Because specificity estimates were inconsistently reported across eligible systematic reviews and several reviews lacked sufficient variance information, only sensitivity could be quantitatively synthesized. Specificity findings were therefore summarized narratively. Six systematic reviews (29, 30, 33, 35, 36, 40) that conducted meta-analyses provided pooled estimates suitable for quantitative synthesis (Table 3). The results of the meta-meta-analysis, employing robust variance estimation to account for overlap, are summarized below and visualized in forest plots (Figure 2). Six studies (29, 30, 33, 35, 36, 40) were included in meta-meta-analysis after removing the overlapping studies. Pooled Sensitivity obtained was 0.90 (95% CI: 0.81–0.99). The I² statistic was 85% indicating substantial heterogeneity between the studies.

**Table 3:** Characteristics of reviews included in the meta-meta-analysis.

Study	Year	Pooled sensitivity	Standard Error	Lower CI	Upper CI	Sample Size	AI Model
Elmakaty et al.	2022	0.90	0.005	0.86	0.93	3,200	Mixed
Kim et al.	2022	0.90	0.004	0.86	0.93	1,900	CNN
Li et al.	2024	0.90	0.187	0.86	0.93	3,015	Mixed
Pirayesh et al.	2024	0.98	0.408	0.96	0.99	2,700	CNN
Sahoo et al.	2024	0.85	0.142	0.82	0.88	11,330	CNN
Ren et al.	2025	0.95	0.265	0.93	0.97	4,800	CNN

### 3.7 Certainty of evidence (GRADE assessment)

The GRADE assessment of the overall body of evidence is summarized in Table 4. The certainty varies substantially across outcomes:

a) Moderate certainty for the diagnostic accuracy of histopathology-based AI. The rating was downgraded from high due to serious inconsistency (high statistical heterogeneity, I^2^ > 75%).
b) Low certainty for the diagnostic accuracy of clinical photography-based AI. Downgraded for inconsistency and for imprecision (wide confidence intervals crossing clinically important thresholds).
c) Very Low certainty for: diagnostic accuracy of OCT-based AI; all outcomes related to implementation feasibility (cost-effectiveness, workflow integration); comparison of AI with conventional methods in real-world settings. These were downgraded for very serious indirectness (limited real-world data), serious imprecision, and often for high risk of bias.

**Table 4:** GRADE summary of findings table.

Outcome	Anticipated Absolute Effects (95% CI)	No. of Reviews (Primary Studies)	Certainty	Outcome
Histopathology-AI for OSCC detection	Sensitivity: 87 patients correctly identified per 100 with OSCC (82 to 91) Specificity: 86 patients correctly identified per 100 without OSCC (81 to 90)	9 (147)	⊕⊕⊕◯ Moderate	AI shows high accuracy but requires biopsy
Clinical Photography-AI for OSCC screening	Sensitivity: 87 patients correctly identified per 100 with OSCC (83 to 90) Specificity: 85 patients correctly identified per 100 without OSCC (80 to 89)	6 (82)	⊕⊕◯◯ Low	Promising for triage but limited real-world validation
OCT-AI for early invasion detection	Sensitivity: 90 patients correctly identified per 100 with OSCC (85 to 94) Specificity: 88 patients correctly identified per 100 without OSCC (82 to 93)	3 (24)	⊕◯◯◯ Very Low	Technically promising but evidence very limited
Superiority of Histopathology-AI over Clinical-AI	DOR: 2.15 times higher diagnostic odds (1.42 to 3.26)	15 (253)	⊕⊕◯◯ Low	Histopathology-AI more accurate but less accessible
Implementation feasibility	Qualitative evidence only	7 (Mixed)	⊕◯◯◯ Very Low	Major barriers identified; minimal real-world implementation

Factors reducing certainty:

(a) Risk of Bias: Many primary studies had high risk of bias in patient selection, index test conduct, or reference standard application, as reported in the included reviews. Additionally, the systematic reviews themselves had variable methodological quality.
(b) Inconsistency: Downgrading for inconsistency was primarily driven by substantial heterogeneity across the included reviews, reflecting differences in AI architectures, imaging modalities, study populations, reference standards, and reported diagnostic performance.
(c) Indirectness: Downgrading for indirectness reflected the predominance of retrospective validation studies, limited multicenter and prospective evidence, insufficient external validation, and the fact that most AI systems were evaluated under controlled research conditions rather than routine clinical practice
(d) Imprecision: Confidence intervals for some estimates were wide and crossed clinically important thresholds (e.g., sensitivity of 90%).
(e) Publication bias: Evidence of funnel plot asymmetry and positive result bias.

## Discussion

### Principal findings and interpretation

This umbrella review represents the most comprehensive synthesis to date of evidence on artificial intelligence for oral squamous cell carcinoma diagnosis, encompassing 15 systematic reviews and 341 primary studies. Our analysis reveals that AI, particularly deep learning models applied to histopathology, achieves promising diagnostic performance (pooled sensitivity: 0.90), with histopathology-based approaches significantly outperforming other modalities. However, these promising accuracy metrics are tempered by several critical findings that challenge the readiness of these technologies for widespread clinical deployment.

First, the evidence exhibits substantial heterogeneity (I^2^ > 85%), with prediction intervals indicating that a new AI application could reasonably have sensitivity anywhere between 71%–96%. This variability stems from differences in AI architectures, training datasets, image modalities, and methodological approaches across studies (44). Second, the methodological quality of systematic reviews in this domain is concerningly variable, with only 20% achieving high confidence according to AMSTAR-2 standards. Common deficiencies include inadequate justification for excluded studies, insufficient risk of bias assessment, and lack of publication bias investigation (45).

Third, we identified moderate redundancy in the evidence base (CCA = 9.06%), with the same seminal primary studies appearing in multiple reviews, creating statistical dependency that is rarely acknowledged or addressed in primary syntheses (46).

Although the pooled estimates suggest that AI demonstrates promising diagnostic performance for OSCC detection, these findings should be interpreted with caution because they are derived from an evidence base with considerable methodological fragility. Only a small proportion of the included systematic reviews achieved high methodological confidence, whereas several were rated as critically low quality according to AMSTAR-2. Deficiencies such as inadequate risk-of-bias assessment, incomplete reporting of excluded studies, and suboptimal meta-analytic methods increase the likelihood of biased summary estimates and reduce confidence in the reported diagnostic performance. Furthermore, the moderate overlap of primary studies indicates that a limited number of influential datasets contributed disproportionately to multiple systematic reviews, potentially amplifying the apparent consistency of findings without providing truly independent evidence (47). Combined with the substantial statistical heterogeneity observed across reviews, these methodological limitations suggest that the pooled estimates should be interpreted as indicators of the current direction of evidence rather than definitive measures of AI performance.

### Clinical implications

Current evidence suggests that artificial intelligence has the potential to function as an adjunctive decision-support tool for the detection of oral squamous cell carcinoma, particularly in histopathological image interpretation and selected image-based screening applications. However, the available evidence is characterized by substantial heterogeneity, variable methodological quality, limited external validation, and a paucity of prospective implementation studies. Therefore, AI should currently be regarded as a complementary aid to, rather than a replacement for, clinician judgment and histopathological confirmation (48). Clinicians must recognize the current limitations: AI performance varies significantly across different implementations, most systems lack robust external validation, and few have been evaluated prospectively in real clinical workflows.

For screening programs and primary care, clinical photography-based AI shows sufficient accuracy for triage applications, potentially extending screening reach in resource-limited settings (49). Mobile implementations offer particular promise but require careful validation in diverse field conditions. OCT-AI demonstrates potential for non-invasive assessment of early invasion but remains largely experimental due to cost and expertise requirements.

### Research implications and future directions

A transition is required from predominantly reporting diagnostic accuracy in controlled environments to evaluating real-world implementation and clinical impact. Future studies should prioritize pragmatic clinical trials comparing AI-assisted diagnosis with standard care, focusing on patient-centered outcomes such as time to treatment initiation, survival rates, and quality of life. Additionally, implementation science approaches using structured frameworks such as RE-AIM and CFIR are essential to systematically identify contextual barriers and facilitators across diverse healthcare settings. Robust health economic evaluations are equally critical to determine cost-effectiveness from both healthcare system and societal perspectives, thereby informing policy and large-scale adoption.

Second, methodological rigor must be strengthened in future systematic reviews. Comprehensive and reproducible search strategies, including grey literature, are necessary to minimize selection bias. Transparent reporting of excluded studies, appropriate risk of bias assessment using validated tools such as QUADAS-2, formal evaluation of publication bias, and adherence to reporting standards like PRISMA-DTA and emerging STARD-AI guidelines will enhance credibility and reliability of synthesized evidence.

Third, greater emphasis on standardization and reproducibility is required to improve comparability and generalizability of findings. Establishing consensus benchmark datasets for OSCC AI research, developing standardized validation protocols and performance metrics, and promoting open science practices—including code, model, and ethically permissible data sharing—will facilitate transparency and replication. Furthermore, fairness-aware model development is essential to ensure equitable performance across different demographic and geographic populations.

Finally, exploration of advanced AI paradigms represents a promising direction. Multimodal AI models integrating histopathological, genomic, clinical, and imaging data could enable more comprehensive disease characterization. Federated learning approaches offer opportunities for collaborative model development while preserving patient data privacy. Incorporation of explainable AI techniques can enhance clinician trust and interpretability, and continuous learning systems may allow AI tools to adapt dynamically to evolving datasets and clinical practices. Collectively, these strategies will move the field beyond proof-of-concept accuracy studies toward clinically meaningful, scalable, and ethically robust AI integration in OSCC care.

### Strengths and limitations

This umbrella review has several notable strengths. Methodologically, we employed a comprehensive search strategy, duplicate review processes, rigorous quality appraisal using AMSTAR-2, quantification of study overlap (CCA), and advanced statistical techniques including robust variance estimation to account for dependency in the meta-meta-analysis. Our application of GRADE to umbrella review evidence represents a methodological advancement in evidence synthesis.

Several limitations must be acknowledged. First, our analysis is at the review level; an individual patient data (IPD) meta-analysis of primary studies would provide stronger evidence but was not feasible given resource constraints. Second, while we quantified overlap using CCA and employed statistical corrections, perfect de-duplication would require extracting all primary study references from 15 reviews—a substantial task. Third, the rapid evolution of AI technology means that primary studies synthesized in included reviews (mostly published before 2023) may not represent the state-of-the-art in 2025. Fourth, publication and language biases may persist despite our efforts to search preprint servers; we included only English-language reviews due to resource limitations. Finally, as with any systematic review, our findings are limited by the quality and completeness of reporting in the included studies. The downgrading of certainty for inconsistency and indirectness reflects important limitations in the current evidence base.

## Conclusion

AI-based approaches demonstrate promising diagnostic performance for OSCC, particularly in histopathological image analysis. However, substantial heterogeneity, limited external validation, and a lack of implementation-focused research currently restrict translation into routine clinical practice. Future studies should prioritize prospective multicenter validation, standardized reporting, and evaluation of real-world clinical impact.

This umbrella review provides a synthesis of available evidence and a strategic roadmap for researchers, clinicians, healthcare administrators, and policymakers. By addressing the identified gaps and methodological deficiencies, we can work toward realizing the transformative potential of AI in reducing the global burden of oral cancer through earlier, more accurate, and more accessible diagnosis.

## Data Availability

The original contributions presented in the study are included in the article/Supplementary Material, further inquiries can be directed to the corresponding author/s.
