# Supplementary material for: Diagnostic performance of artificial intelligence in oral squamous cell carcinoma detection: a higher-order evidence synthesis through umbrella review and meta-meta-analysis
Source: Front Dent Med. 2026 Sep 8;7:1915105. doi: 10.3389/fdmed.2026.1915105 (PMC13593500; doi:10.3389/fdmed.2026.1915105)
Supplement: Supplementary file 1 [file Supplementaryfile1.docx]

**Supplementary File S1
Complete Database Search Strategies**

# 1. MEDLINE/PubMed

#1 Oral cancer
("Mouth Neoplasms"[Mesh] OR "Mouth Neoplasms"[Title/Abstract] OR "oral cancer"[Title/Abstract] OR "oral cancers"[Title/Abstract] OR "oral carcinoma"[Title/Abstract] OR "oral squamous cell carcinoma"[Title/Abstract] OR "oral squamous cell carcinomas"[Title/Abstract] OR OSCC[Title/Abstract] OR "oral cavity cancer"[Title/Abstract] OR "oral cavity carcinoma"[Title/Abstract] OR "tongue cancer"[Title/Abstract] OR "tongue neoplasms"[Title/Abstract])

#2 Artificial intelligence
("Artificial Intelligence"[Mesh] OR "Machine Learning"[Mesh] OR "Neural Networks, Computer"[Mesh] OR "artificial intelligence"[Title/Abstract] OR "machine learning"[Title/Abstract] OR "deep learning"[Title/Abstract] OR "neural network*"[Title/Abstract] OR "convolutional neural network*"[Title/Abstract] OR CNN[Title/Abstract] OR CNNs[Title/Abstract] OR "support vector machine*"[Title/Abstract] OR SVM[Title/Abstract] OR "computer-aided diagnosis"[Title/Abstract] OR "computer-assisted diagnosis"[Title/Abstract] OR "automated diagnosis"[Title/Abstract])

#3 Systematic reviews/meta-analyses
("Systematic Review"[Publication Type] OR "Meta-Analysis"[Publication Type] OR "systematic review"[Title/Abstract] OR "systematic reviews"[Title/Abstract] OR "meta-analysis"[Title/Abstract] OR "meta analysis"[Title/Abstract] OR "meta-analyses"[Title/Abstract] OR "umbrella review"[Title/Abstract] OR "review of reviews"[Title/Abstract])

#4 Final search
#1 AND #2 AND #3

Coverage: Database inception to September 30, 2025.

# 2. Embase via Ovid

1. exp mouth cancer/ OR exp oral cavity cancer/ OR exp tongue cancer/ OR (oral cancer or oral cancers or oral carcinoma or oral squamous cell carcinoma or oral squamous cell carcinomas or OSCC or oral cavity cancer or oral cavity carcinoma or tongue cancer).ti,ab,kw.

2. exp artificial intelligence/ OR exp machine learning/ OR exp deep learning/ OR exp neural network/ OR exp computer assisted diagnosis/ OR (artificial intelligence or machine learning or deep learning or neural network* or convolutional neural network* or CNN or CNNs or support vector machine* or SVM or computer-aided diagnosis or computer-assisted diagnosis or automated diagnosis).ti,ab,kw.

3. exp systematic review/ OR exp meta analysis/ OR (systematic review or systematic reviews or meta-analysis or meta analysis or meta-analyses or umbrella review or review of reviews).ti,ab,kw.

4. 1 AND 2 AND 3

Coverage: Database inception to September 30, 2025.

# 3. Scopus

TITLE-ABS-KEY(
"oral cancer" OR "oral cancers" OR "oral carcinoma" OR "oral squamous cell carcinoma" OR "oral squamous cell carcinomas" OR OSCC OR "oral cavity cancer" OR "oral cavity carcinoma" OR "tongue cancer"
)
AND
TITLE-ABS-KEY(
"artificial intelligence" OR "machine learning" OR "deep learning" OR "neural network*" OR "convolutional neural network*" OR CNN OR CNNs OR "support vector machine*" OR SVM OR "computer-aided diagnosis" OR "computer-assisted diagnosis" OR "automated diagnosis"
)
AND
TITLE-ABS-KEY(
"systematic review" OR "systematic reviews" OR "meta-analysis" OR "meta analysis" OR "meta-analyses" OR "umbrella review" OR "review of reviews"
)

Coverage: Database inception to September 30, 2025.

# 4. Web of Science Core Collection

TS=(
"oral cancer" OR "oral cancers" OR "oral carcinoma" OR "oral squamous cell carcinoma" OR "oral squamous cell carcinomas" OR OSCC OR "oral cavity cancer" OR "oral cavity carcinoma" OR "tongue cancer"
)
AND
TS=(
"artificial intelligence" OR "machine learning" OR "deep learning" OR "neural network*" OR "convolutional neural network*" OR CNN OR CNNs OR "support vector machine*" OR SVM OR "computer-aided diagnosis" OR "computer-assisted diagnosis" OR "automated diagnosis"
)
AND
TS=(
"systematic review" OR "systematic reviews" OR "meta-analysis" OR "meta analysis" OR "meta-analyses" OR "umbrella review" OR "review of reviews"
)

Coverage: Database inception to September 30, 2025.

# 5. Cochrane CENTRAL

#1 MeSH descriptor: [Mouth Neoplasms] explode all trees

#2 ("oral cancer" OR "oral cancers" OR "oral carcinoma" OR "oral squamous cell carcinoma" OR "oral squamous cell carcinomas" OR OSCC OR "oral cavity cancer" OR "oral cavity carcinoma" OR "tongue cancer"):ti,ab,kw

#3 #1 OR #2

#4 MeSH descriptor: [Artificial Intelligence] explode all trees

#5 ("artificial intelligence" OR "machine learning" OR "deep learning" OR "neural network*" OR "convolutional neural network*" OR CNN OR CNNs OR "support vector machine*" OR SVM OR "computer-aided diagnosis" OR "computer-assisted diagnosis" OR "automated diagnosis"):ti,ab,kw

#6 #4 OR #5

#7 ("systematic review" OR "systematic reviews" OR "meta-analysis" OR "meta analysis" OR "meta-analyses" OR "umbrella review" OR "review of reviews"):ti,ab,kw

#8 #3 AND #6 AND #7

# CONCEPT TABLE

| PICOS component | Entry terms / synonyms / related terms |
| --- | --- |
| P – Population | Oral Squamous Cell Carcinoma; Oral Squamous Cell Carcinomas; OSCC; Oral Cancer; Oral Cancers; Oral Carcinoma; Oral Carcinomas; Oral Cavity Cancer; Oral Cavity Carcinoma; Mouth Cancer; Mouth Neoplasm; Mouth Neoplasms; Oral Neoplasm; Oral Neoplasms; Tongue Cancer; Tongue Carcinoma; Tongue Neoplasm; Tongue Neoplasms; Head and Neck Cancer*; Head and Neck Squamous Cell Carcinoma; HNSCC |
| I – Index test | Artificial Intelligence; AI; Machine Learning; ML; Deep Learning; DL; Neural Network*; Artificial Neural Network*; ANN; Convolutional Neural Network*; CNN; CNNs; Deep Neural Network*; DNN; Support Vector Machine*; SVM; Random Forest*; k-Nearest Neighbor*; k-NN; Computer Vision; Automated Image Analysis; Image Classification; Image Recognition; Computer-Aided Diagnosis; Computer-Assisted Diagnosis; Computer-Aided Detection; Automated Diagnosis; Artificial Intelligence-Assisted Diagnosis; AI-Assisted Diagnosis; AI-Based Diagnosis; AI-Based Detection |
| I – Diagnostic modality | Histopathology; Histopathological Image*; Histology; Histological Image*; Digital Histopathology; Digital Pathology; Whole-Slide Image*; WSI; Tissue Microarray*; TMA; Clinical Image*; Clinical Photograph*; Oral Photograph*; Smartphone Image*; Optical Coherence Tomography; OCT; Autofluorescence; Autofluorescence Imaging; Cytology; Cytological Image*; Radiography; Radiographic Image*; Panoramic Radiograph*; Orthopantomogram*; OPG; Cone-Beam Computed Tomography; CBCT; Hyperspectral Imaging |
| C – Comparator / Reference standard | Histopathology; Histopathological Diagnosis; Histological Diagnosis; Histological Examination; Histopathological Examination; Biopsy; Tissue Biopsy; Surgical Biopsy; Incisional Biopsy; Excisional Biopsy; Reference Standard; Gold Standard; Clinical Examination; Visual Examination; Visual-Tactile Examination; Conventional Diagnosis; Conventional Diagnostic Method*; Expert Diagnosis; Pathologist Diagnosis |
| O – Outcome | Diagnostic Accuracy; Diagnostic Performance; Test Accuracy; Sensitivity; Specificity; Accuracy; Area Under the Curve; Area Under the Receiver Operating Characteristic Curve; AUC; AUROC; Receiver Operating Characteristic; ROC; ROC Curve; Diagnostic Odds Ratio; DOR; Positive Likelihood Ratio; PLR; Negative Likelihood Ratio; NLR; Positive Predictive Value; PPV; Negative Predictive Value; NPV; True Positive; True Negative; False Positive; False Negative; Detection Rate; Classification Performance |
| S – Study design | Systematic Review; Systematic Reviews; Meta-Analysis; Meta-Analyses; Meta Analysis; Meta Analyses; Systematic Review and Meta-Analysis; Systematic Review and Meta Analysis; Quantitative Review; Evidence Synthesis; Research Synthesis; Review of Reviews; Umbrella Review; Overview of Reviews; Systematic Review of Reviews; Systematic Overview |
