# Supplementary material for: Diagnostic performance of artificial intelligence in oral squamous cell carcinoma detection: a higher-order evidence synthesis through umbrella review and meta-meta-analysis
Source: Front Dent Med. 2026 Sep 8;7:1915105. doi: 10.3389/fdmed.2026.1915105 (PMC13593500; doi:10.3389/fdmed.2026.1915105)
Supplement: Supplementary file 2 [file Supplementaryfile2.docx]

AMSTAR-2 RISK OF BIAS ASSESSMENT

1. Overview of Assessment

The methodological quality of the 15 included systematic reviews was independently assessed by two reviewers using the AMSTAR-2 (A MeaSurement Tool to Assess systematic Reviews 2) checklist. Each review was evaluated against 16 domains, with particular attention to 7 critical domains that are essential for the validity of a systematic review's conclusions. Inter-rater agreement was high (Cohen's κ = 0.86), with discrepancies resolved through consensus discussion.

2. Critical Domain Analysis

Supp. Table 1: Performance on Critical AMSTAR-2 Domains

| Critical Domain | Description | No. of Reviews Meeting Criterion (%) | Key Issues Identified |
| --- | --- | --- | --- |
| Item 2: Duplicate study selection and data extraction | Did the review authors perform study selection in duplicate? | 15 (100%) | All reviews reported duplicate selection |
| Item 4: Comprehensive literature search | Did the review authors perform a comprehensive literature search? | 15 (100%) | All reported comprehensive search strategies |
| Item 7: List and justify excluded studies | Did the review authors provide a list of excluded studies and justify the exclusions? | 2 (13.3%) | MAJOR DEFICIENCY: 13 reviews provided no excluded study list |
| Item 9: Risk of bias assessment of included studies | Did the review authors use a satisfactory technique for assessing the risk of bias (RoB) in individual studies? | 6 (40.0%) | MAJOR DEFICIENCY: 9 reviews used inadequate or no RoB tools |
| Item 11: Appropriate methods for meta-analysis | If meta-analysis was performed, did the review authors use appropriate methods? | 8/10 meta-analyses (80.0%) | 2 reviews used inappropriate statistical methods |
| Item 13: Consideration of RoB in interpretation | Did the review authors account for RoB in individual studies when interpreting results? | 10 (66.7%) | 5 reviews did not consider RoB in conclusions |
| Item 15: Assessment of publication bias | Did the review authors investigate publication bias? | 5 (33.3%) | MAJOR DEFICIENCY: 10 reviews did not assess publication bias |

3. Review-Specific Critical Appraisal

3.1 High Confidence Reviews (n=3)

Elmakaty (2022) - Critical Reviews in Oncology/Hematology

- Strengths: Used QUADAS-2 appropriately, conducted comprehensive meta-analysis, assessed publication bias, considered RoB in conclusions
- Weaknesses: Did not report funding sources for included studies
- Overall: Exemplary methodology with minor reporting omissions

Kim (2022) - Cancers

- Strengths: Robust QUADAS-2 application, appropriate meta-analysis methods, investigation of heterogeneity and publication bias
- Weaknesses: Limited explanation of study design choices
- Overall: High methodological rigor with comprehensive reporting

Sahoo (2024) - Frontiers in Oral Health

- Strengths: Large-scale review (55 studies), appropriate use of QUADAS-2, thorough heterogeneity assessment
- Weaknesses: No funding source reporting
- Overall: Comprehensive review with minor reporting deficiencies

3.2 Moderate Confidence Reviews (n=5)

Alabi (2021) - Artificial Intelligence in Medicine

- Critical Weakness: Used PROBAST tool but application was partial
- Strengths: Comprehensive search, duplicate processes, appropriate synthesis
- Overall: Good methodology but RoB assessment could be improved

Khanagar (2023) - Biomedicines

- Critical Weakness: QUADAS-2 application was adequate but not comprehensive
- Strengths: GRADE framework used, appropriate meta-analysis
- Overall: Solid methodology with room for improvement in RoB assessment

Li (2024) - International Journal of Surgery

- Critical Weakness: Risk of bias tool not clearly specified
- Strengths: Comprehensive methods, appropriate statistical approaches
- Overall: Methodologically sound but reporting transparency issues

Nieri (2025) - Oral Diseases

- Critical Weakness: QUADAS-C used but exclusion rationale weak
- Strengths: Comparative design, comprehensive search
- Overall: Innovative design with methodological limitations

Pirayesh (2024) - Journal of Oral Pathology and Medicine

- Critical Weakness: QUADAS-2 application could be more rigorous
- Strengths: Strong meta-analysis, comprehensive methods
- Overall: Good quality with minor methodological concerns

3.3 Low Confidence Reviews (n=4)

Chowdhury (2025) - Cureus

- Critical Flaws: No excluded study list; inadequate RoB assessment
- Strengths: Comprehensive search, duplicate processes
- Overall: Limited confidence due to major methodological deficiencies

Khanagar (2021) - Diagnostics

- Critical Flaws: Weak RoB assessment; no excluded study list
- Strengths: Comprehensive search strategy
- Overall: Major limitations in critical appraisal methods

Mahmood (2020) - Oral Oncology

- Critical Flaws: "Tailored" QUADAS-2 without validation; no excluded study list
- Strengths: Early comprehensive review
- Overall: Methodological innovations but insufficient rigor

Ren (2025) - Journal of Stomatology, Oral and Maxillofacial Surgery

- Critical Flaws: Weak exclusion rationale; questionable meta-analysis methods
- Strengths: Large number of included studies
- Overall: Quantity over quality approach

3.4 Critically Low Confidence Reviews (n=3)

Jerjes (2024) - Journal of Clinical Medicine

- Multiple Critical Flaws: No specified RoB tool; inadequate synthesis methods; did not consider RoB in conclusions
- Additional Issues: Protocol not fully registered; no funding reporting
- Overall: Serious methodological limitations throughout

Malhotra (2024) - Asian Pacific Journal of Cancer Prevention

- Multiple Critical Flaws: No protocol; poor diagnostic accuracy meta-analysis methods; no RoB consideration in conclusions
- Additional Issues: No excluded study list; no funding reporting
- Overall: Fundamentally flawed methodology

Thakuria (2024) - Expert Review of Medical Devices

- Multiple Critical Flaws: Narrative quality appraisal only; no systematic RoB assessment; no meta-analysis despite appropriate data
- Additional Issues: Protocol issues; partial search strategy
- Overall: More narrative than systematic review

4. Impact of Quality on Results

Supp Table 2: Association Between AMSTAR-2 Rating and Reported Accuracy

| AMSTAR-2 Rating | Number of Reviews | Average Reported Sensitivity Range | Average Reported Specificity Range | Consistency of Findings |
| --- | --- | --- | --- | --- |
| High | 3 | 0.87-0.95 | 0.81-0.97 | High consistency, narrow ranges |
| Moderate | 5 | 0.89-1.00 | 0.57-0.99 | Moderate consistency |
| Low | 4 | 0.70-1.00 | 0.57-1.00 | Wide variability |
| Critically Low | 3 | 0.43-1.00 | 0.50-1.00 | Extremely wide variability |

Key Finding: Higher quality reviews reported more conservative and consistent accuracy estimates, while lower quality reviews showed wider, more optimistic ranges.

6. Temporal Trends in Methodological Quality

Supp. Table 3: Quality Trends by Publication Year

| Publication Period | High | Moderate | Low | Critically Low | Average Quality |
| --- | --- | --- | --- | --- | --- |
| 2020-2021 (n=3) | 0 | 1 | 2 | 0 | Low-Moderate |
| 2022 (n=2) | 2 | 0 | 0 | 0 | High |
| 2023 (n=1) | 0 | 1 | 0 | 0 | Moderate |
| 2024 (n=6) | 1 | 2 | 1 | 2 | Mixed |
| 2025 (n=3) | 0 | 1 | 1 | 1 | Low |

Observation: No clear temporal improvement in methodological quality, with 2024-2025 reviews showing the widest quality variation.

7. Most Common Methodological Deficiencies

Supp. Table 4: Frequency of Major Deficiencies Across All Reviews

| Deficiency | Number of Reviews (%) | Impact on Review Validity |
| --- | --- | --- |
| No list/justification of excluded studies | 13 (86.7%) | High - Limits reproducibility |
| Inadequate risk of bias assessment | 9 (60.0%) | Critical - Undermines confidence in conclusions |
| No investigation of publication bias | 10 (66.7%) | High - Risk of overestimated effects |
| No reporting of funding sources | 11 (73.3%) | Moderate - Limits assessment of conflicts |
| Incomplete study design explanation | 13 (86.7%) | Moderate - Affects interpretation |
| Poor meta-analysis methods | 2/10 (20.0%) | Critical - Invalid quantitative synthesis |

8. Domain-Specific Recommendations for Future Reviews

Based on our assessment, we recommend future systematic reviews on AI in OSCC diagnosis prioritize:

1. Mandatory reporting of excluded studies with justifications (Item 7)
2. Rigorous application of validated RoB tools like QUADAS-2 (Item 9)
3. Systematic investigation of publication bias (Item 15)
4. Transparent reporting of funding sources for all included studies (Item 10)
5. Protocol registration before review commencement (Item 1)
6. Appropriate meta-analysis methods for diagnostic accuracy data (Item 11)

9. Limitations of Our Assessment

Our AMSTAR-2 assessment has several limitations:

- Some judgments required interpretation of incompletely reported methods
- We assumed best practices when methods were ambiguously described
- The tool itself has limitations for diagnostic test accuracy reviews
- Assessment of some domains (e.g., comprehensive search) relied on author reporting

10. Implications for Evidence Synthesis

The variable methodological quality has important implications:

- High and Moderate quality reviews can be weighted more heavily in evidence synthesis
- Low and Critically Low quality reviews require cautious interpretation
- Quantitative synthesis should consider quality stratification
- Future updates should prioritize inclusion of higher quality reviews
